# Tophaceous Gout of the Patella: A Report of Two Cases

**DOI:** 10.1155/2012/253693

**Published:** 2012-11-07

**Authors:** Graeme Hopper, Sanjay Gupta, Sarath Bethapudi, David Ritchie, Elaine MacDuff, Ashish Mahendra

**Affiliations:** ^1^Trauma and Orthopaedics, Glasgow Royal Infirmary, 84 Castle Street, Glasgow G4 0SF, UK; ^2^Radiology, Glasgow Western Infirmary, Dumbarton Road, Glasgow G11 6NT, UK; ^3^Pathology, Glasgow Western Infirmary, Dumbarton Road, Glasgow G11 6NT, UK

## Abstract

*Introduction*. Tophaceous gout of the patella is rare and may masquerade as a tumour or tumour-like condition. *Cases*. We report two patients with gout involving the patella, one complicated by a pathological fracture and the other occurring in a bipartite patella in a young adult. *Discussion*. Typical imaging appearances and measurement of serum urate will usually confirm the diagnosis but, occasionally, the serum urate level may be normal in active gout and in such cases, a biopsy will be required. *Conclusion*. Gout of the patella may masquerade as a tumour or tumour-like condition and it is important to consider gout in the differential diagnosis.

## 1. Introduction

Gout is a relatively common crystal arthropathy caused by chronic hyperuricaemia resulting in the deposition of monosodium urate crystals around joints and tendons. In chronic tophaceous gout, crystals may be deposited in tendons and may cause osseous erosions at their bony attachments. Involvement of the extensor tendon of the knee is not an uncommon site for gout but involvement of the patella is rare and underreported in the literature [1–13]. For patients who present with a patellar lesion, the diagnosis of gout might not be suspected and patients given a provisional diagnosis of infection, tumour, metabolic disease, and degenerative or inflammatory arthropathy. We report two patients with gout involving the patella and discuss the clinical, imaging, and pathological features, differential diagnosis, and management. Patients gave informed consent prior to inclusion in this paper and each author certifies that there is no actual or potential conflict of interests in relation to this paper.

## 2. Case Reports

### 2.1. Case 1

**Figure 1:**
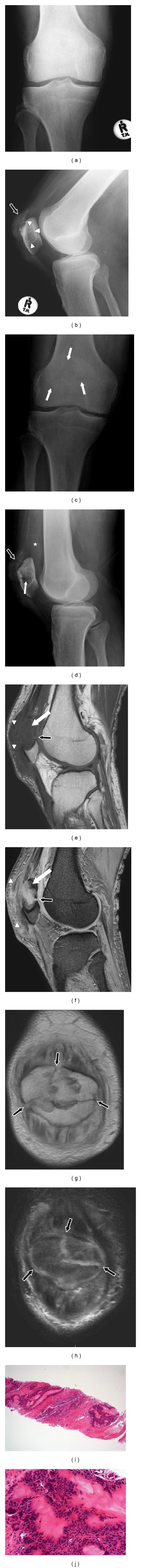
(a) AP and (b) lateral radiographs of right knee demonstrate diffuse faint calcification involving the insertion of the quadriceps tendon at the upper pole of the patella (black arrow). Note the well-defined lytic lesion with marginal sclerosis (arrowheads) in the patella and prepatellar soft tissue swelling. (c) AP and (d) lateral radiographs of the right knee a year later show a comminuted pathological fracture of the mid patella involving the lytic lesion (white arrows) with associated joint effusion (star). The faint calcification within the distal quadriceps is again noted (black arrow). Sagittal (e) T1-WSE and (f) T2-WGE and coronal (g) PDSE and (h) STIR MR images confirm a comminuted pathological fracture of the mid and upper patella (black arrows) involving the patellar lesion (white arrows) that displays nonspecific features. However, there is a prominent inhomogeneous soft tissue mass in the prepatellar region, some of which displays low signal intensity (SI) on all sequences that corresponds to the mineralisation noted on the radiographs (arrowheads). (i) Core biopsy at low power and (j) core biopsy at high power, stained with H&E. There are aggregates of amorphous eosinophilic material surrounded by a palisade of histiocytes and giant cells in keeping with tophaceous gout.

A 70-year-old gentleman presented to his general practitioner with a two-month history of pain and swelling involving the anterior aspect of his right knee. He had no past medical history of note and no history of arthropathy or predisposing factors of gout. Radiographs of his right knee showed a faintly and partially mineralised soft tissue mass mainly overlying the superior aspect of the patella as well as a well-defined lytic lesion with marginal sclerosis in the mid patella (Figures 1(a) and 1(b)). Unfortunately, the patient was lost to follow up until he represented to the accident and emergency a year later, having injured his right knee. Radiographs of the knee showed a slightly displaced, comminuted pathological fracture of the patella as well as the previously noted partially mineralised soft tissue mass overlying the patella. The fracture was treated conservatively with a splint (Figures 1(c) and 1(d)). MR imaging confirmed the pathological fracture as well as the prominent prepatellar mass infiltrating the distal quadriceps and proximal patellar tendons (Figures 1(e)–1(h)). The elevated serum urate levels (1.04 mmol/L, normal range <0.40 mmol/L) confirmed active gout but a biopsy was felt justified in view of the unusual site and the need to exclude a coexisting tumour. Subsequent histological examination revealed aggregates of amorphous eosinophilic material surrounded by a palisade of histiocytes and giant cells in keeping with tophaceous gout (Figures 1(i) and 1(j)). This gentleman was referred back to his GP for appropriate medical management of gout with colchicine and allopurinol. He was reviewed one year later in the oncology clinic, his symptoms had improved significantly, and radiographs demonstrated satisfactory healing of his fracture.

### 2.2. Case 2

**Figure 2:**
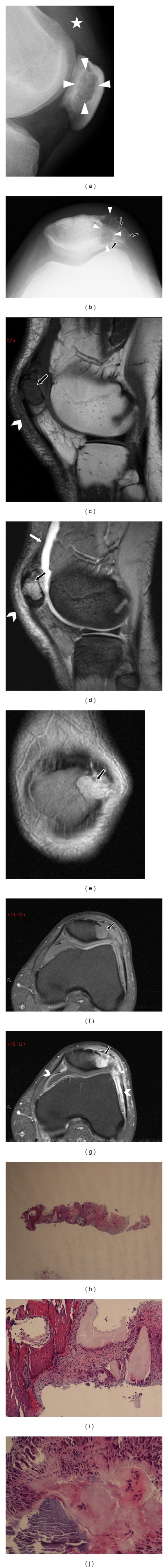
(a) Lateral and (b) sky line view radiographs of the patella demonstrate a well-defined slightly expansile lytic lesion with sclerotic margin in the mid lateral aspect of the patella (arrowheads) and knee joint effusion (star). On the skyline view, there is also evidence of a bipartite patella (black arrows). Sagittal (c) T1-WSE and (d) T2-WGE and (e) coronal T2-WSE MR images demonstrate a well-defined mass in the patella (black arrows) that displays intermediate SI on T1-W and moderately increased SI on T2-W MR images. Note the prepatellar bursitis (chevrons) and reactive joint effusion (white arrow). Axial T1-WSE fat saturated (f) before and (g) after gadolinium MRI images, show avid enhancement of the patellar lesion (black arrows), involvement of the adjacent lateral parapatellar soft tissues and lateral retinaculum (arrowhead), and enhancing reactive synovitis (chevrons). (h) Core biopsy at low power, (i) core biopsy at medium power, and (j) core biopsy at high power stained with H&E. There are aggregates of amorphous eosinophilic material with a surrounding foreign body giant cell and histiocyte reaction consistent with gout.

A 34-year-old gentleman presented to the orthopaedic department with a two-month history of anterior knee pain and swelling involving his left knee. He had no past medical history of note, no history of arthritis or predisposing factors of gout such as obesity, high blood pressure, high cholesterol levels, and heavy alcohol use. Radiographs of his left knee showed a well-defined slightly expansile, lytic lesion involving the patella (Figures 2(a) and 2(b)). MR imaging showed a slightly inhomogeneous lesion involving the superolateral aspect of the patella with soft tissue infiltration around the patellar attachment of the lateral retinaculum (Figures 2(c)–2(g)). Serum urate levels were slightly raised at 0.54 mmol/L. An ultrasound-guided biopsy of this lesion was subsequently arranged to confirm the diagnosis. Histological examination revealed amorphous material consistent with gout (Figures 2(h) and 2(i)). This gentleman was referred back to his GP for appropriate medical management of gout with colchicine and allopurinol and has had no reported adverse outcomes.

## 3. Discussion

Tophaceous gout of the patella was first reported in 1955 by Peloquin [10]. They presented a patient who was found to have erosion of the patellar cortex at surgery. Greenberg described the first reported pathological fracture of the patella due to underlying gout in 1986 [5]. Further pathological fractures of the patella due to gout have been published in [1, 2, 11]. Other reports include a nonunion of a patellar fracture as a result of gout [4] and a painful bipartite patella secondary to a gouty tophus [3]. Recht et al. reported seven patients with gouty tophi of the patella and concluded that osteolytic lesions of the superolateral portion of the patella with an associated soft tissue mass should raise the possibility of gout [13]. Reber et al. reported three cases of patellar gout, one in a bipartite patella [12].

The classical history and examination findings of gout include previous attacks of gouty arthritis, tophaceous deposits, swelling of the joint, and occasional pain. Our reported patients did not have any history of gout but presented with a two-month history of pain and swelling involving the knee. Routine blood tests should include evaluation of serum urate and raised levels will confirm the diagnosis. However, occasionally, the serum urate may be normal during acute gout and in such cases evaluation of synovial fluid or biopsy may be required [14]. Radiographs of the extensor complex of the knee may show soft tissue swelling and mineralisation as in Case 1 but involvement of the patella is rare and raises the possibility of other pathology including tumours, infection, other arthropathies, and metabolic disorders [7–9, 15]. Other arthropathies, including rheumatoid arthritis and seronegative inflammatory arthropathies, osteoarthritis, and calcium pyrophosphate disease are often associated with subarticular changes including geodes and cysts but neither patient gave a history of a known arthropathy and there was no other evidence of arthropathy on the imaging studies of the affected knee joints. The differential also includes bone tumours but tumour-like lesions of the patella are more common than tumours and benign tumours more common than malignant tumours. In patients less than 40 years of age, chondroblastoma and giant cell tumour of bone are the most common diagnoses whereas over the age of 40 years, the most common diagnoses are gout and metastatic disease [15]. Radiologically, there is often difficulty in differentiating benign from malignant lesions. The relative small size of the patella, lack of physeal zones, and absence of periosteum make radiological assessment more difficult. Mineralisation in a patellar lesion may be found with gout and various neoplasms including chondroblastoma, osteoid osteoma, and osteoblastoma, osteosarcoma but these tumours tend to occur in the second decades and gout would be most unusual in this age group unless there was a history of renal disease or genetic disorders associated with hyperuricaemia. A focal lesion in the patella may also be due to acute haematogenous osteomyelitis but this again is usually found in the immature patella and is very rare in adulthood [15]. An expansile lytic lesion should suggest either a giant cell tumour or brown tumour due to hyperparathyroidism and both of these diagnoses were considered in Case 2. Gout in a patient of 34 years is unusual and may be associated with obesity, high blood pressure, high cholesterol levels, and heavy alcohol abuse but none of these predisposing factors were present. Although the serum urate level was slightly elevated in Case 2, a biopsy was felt justified in view of the patient's relatively young age. Soft tissue involvement may occur with malignant lesions, including metastases, lymphomas, and sarcomas as well as gout but the presence of mineralisation within the soft tissue mass adjacent to the patella and in particular, the extensor tendon is more suggestive of gout. Furthermore, the marginal sclerosis around the intrapatellar lesions on the radiographs is a benign feature in keeping with a slow growing lesion as opposed to a malignant lesion that would usually have a more ill-defined zone of transition between lesion and normal bone. Pigmented villo-nodular synovitis (PVNS) and synovial osteochondromatosis (SOC) could also be considered in the differential diagnosis as these conditions may involve tendon sheaths and can cause intraosseous erosions with marginal sclerosis. However, mineralisation is not a feature of PVNS and involvement of the extensor tendon of the knee in SOC is very rare. Cross-sectional imaging may be helpful in lesion characterisation and staging. CT is very sensitive at detecting mineralisation and cortical destruction and MR imaging is the most accurate imaging technique for assessing soft tissue extent. The MR signal characteristics of gouty tophi are nonspecific with low/intermediate signal intensity (SI) on T1-weighting and variable SI on T2-weighting. Low SI foci on T2-weighting may be due to haemosiderin deposition or mineralisation, as in Case 1. Although the serum urate level was also elevated in Case 1, a biopsy was felt justified in view of the unusual site and the need to exclude a coexisting tumour. 

The pathological features of tophaceous gout are characteristic. Macroscopic examination reveals a nodular chalky appearance. Polarised microscopy of a fresh imprint demonstrates positively birefringent needle shaped crystals. These crystals are lost during processing for H&E examination. Instead, histologically, there are nodular aggregates of amorphous material surrounded by a palisade of histiocytes and multinucleated giant cells.

In conclusion, gout of the patella is a rare but recognised condition. We have presented two cases, one complicated by a pathological fracture and other occurring in bipartite patella in a male of 34 years. Gout of the patella may masquerade as a tumour or tumour-like condition and it is important to consider gout in the differential diagnosis. In patients with raised serum urate levels and typical imaging appearances, a biopsy is probably not required but, occasionally, serum urate may be normal in active gout and a cytological or histological proof will be required in such cases.
